# Supplementary material for: Duplex DNA and BLM regulate gate opening by the human TopoIIIα-RMI1-RMI2 complex
Source: Nat Commun. 2022 Jan 31;13:584. doi: 10.1038/s41467-022-28082-5 (PMC8803869; doi:10.1038/s41467-022-28082-5)
Supplement: Supplementary file 1 — Supplementary Information [file 41467_2022_28082_MOESM1_ESM.pdf]

Supplementary Information for:

Duplex DNA and BLM regulate gate opening by the human TopoIIIα-RMI1-RMI2 complex

Julia A.M. Bakx[1#], Andreas S. Biebricher[1#], Graeme A. King[1,2#], Panagiotis Christodoulis[1], Kata Sarlós[3], Anna H. Bizard[3], Ian D. Hickson[3], Gijs J.L. Wuite[1□], Erwin J.G. Peterman[1*□]*

[1] *Department of Physics and Astronomy, and LaserLaB Amsterdam, Vrije Universiteit Amsterdam, De Boelelaan 1081, 1081 HV, Amsterdam, The Netherlands*

[2] *Current address: Institute of Structural and Molecular Biology, University College London, Gower Street, London WC1E 6BT, UK*

[3] *Center for Chromosome Stability and Center for Healthy Aging, Department of Cellular and Molecular Medicine, University of Copenhagen, Blegdamsvej 3B, 2200 Copenhagen N, Denmark*

[#] *These authors contributed equally*

[□] *These authors jointly supervised this work*

**Correspondence should be addressed to G.J.L. Wuite (g.j.l.wuite@vu.nl), E.J.G. Peterman (e.j.g.peterman@vu.nl)*

Supplementary Notes

Supplementary Note 1: TRR-induced length increase of ssDNA requires backbone cleavage

Unlike dsDNA, where the contour length can often increase as a result of binding by proteins / ligands (e.g., RecA, RAD51, intercalators), there is no protein known that significantly increases the ssDNA contour length (0.57 nm/nt) upon binding. This is because ssDNA is non-helical and thus, in contrast to dsDNA, cannot easily be lengthened through unwinding of the backbone. There are reports that ssDNA is able to undergo lengthening of up to 0.7 nm/nt under high forces (>>100 pN)[1,2]. Nonetheless, even this would only result in an additional length of ~23%, much less than the ~50% lengthening of ssDNA we measure for maximum TRR coverage. This indicates that the increase in ssDNA length due to bound TRR cannot be explained without considering local backbone cleavage and gate opening of the protein.

Supplementary Note 2: Characterization of the gate opening equilibrium

We posit that, even at low tensions (<5 pN), the gate opening equilibrium is significantly shifted towards the open gate state for the following reasons. First, no additional lengthening is observed from 5 to 15 pN (Fig. 2c), suggesting that by 5 pN, most of the gates have already opened. Second, if a large fraction of gates were closed at low tensions, we would expect that these would progressively open as the tension is increased, leading to a substantial hysteresis between the forward and backward FD-curves. However, only a small hysteresis is observed (Supplementary Fig. 2e). Finally, given that the footprint of TRR on ssDNA is ~20 nt[3], we anticipate that up to ~1,600 proteins can bind to λ-ssDNA (48,502 nt). This estimation is based on the assumption that, under maximum coverage conditions, a non-cooperative protein with a footprint of 20 nt will be separated from a neighbouring protein by between 0 and 19 nt. Therefore, the randomly distributed regions of bare ssDNA will be, on average, ~10 nt, resulting in an effective footprint of ~30 nt on the DNA. If all of these proteins would be in an open state with a gate size of ~8.5 nm (Fig. 2d), we would expect a maximum lengthening of ~13.6 µm (1600 x 8.5 nm), which is in good agreement with our average lengthening of ~13.3 µm. Together, these observations suggest that most gates are in the open state, from at least 5 pN.

Supplementary Note 3: Interpretation of 'subtraction plots'

Care must be taken when interpreting 'subtraction plots' (such as those shown in Fig. 2c) at forces below ~5 pN. The subtraction plots yield a direct read-out of TRR-ssDNA gate opening only under the assumption that the difference between the corresponding FD-curves is solely caused by changes in the gate size. This is a valid assumption at forces above 5 pN (since TRR binding induces a clear lateral shift in the ssDNA length), but it is unclear whether this assumption still holds at forces below 5 pN for the following reasons. First, it is difficult to rule out that TRR binding induces ssDNA compaction at low forces (in addition to gate opening). Such compaction (resulting in a reduced lengthening) could be erroneously interpreted as gate closing. An additional complication stems from the fact that, in the absence of protein binding, ssDNA will form hairpin structures in standard buffer at low forces, resulting in compaction of the tethered substrate. We expect that TRR binding will reduce the extent of hairpin formation, resulting in a perturbation to the FD-curve of ssDNA, independent of gate opening. Finally, while our optical tweezers instrument is highly sensitive in measuring changes in force (with a precision better than 1 pN), it is less sensitive in measuring absolute forces (precision ~1-2 pN). The latter is particularly relevant at low forces because small differences in the absolute force can yield relatively large changes in the apparent subtraction plot. For these reasons, we currently consider only forces >5 pN as reliable in the interpretation of the subtraction plots.

Supplementary Note 4: Estimating the fraction of open TRR-ssDNA gates in the absence of tension

Mills et al. have characterized the force dependence of the *Ec*TopoI-ssDNA gate opening equilibrium, which consists of two consecutive steps: ssDNA cleavage/religation and gate opening/closing[4]. The total probability that the gates are open (P_{O}) is thus given by the product of the probabilities of ssDNA cleavage (P_{c}) and gate opening (P_{o}), i.e., $P_{\mathrm{O}} = P_{\mathrm{c}} * P_{\mathrm{o}}$. Mills et al. showed that only the gate closing rate is force dependent, with a transition state length (Δx) of ~2.2 nm. If we assume that the force dependence of TRR is similar to that of *Ec*TopoI (based on the fact that both proteins share a similar sequence[5,6]), we can neglect ssDNA cleavage and religation from our consideration (i.e., we assume that $P_{\mathrm{c}} \approx 100\%$). The force-dependent probability of gate opening can then be estimated as[4]:

$$P_{\mathrm{O}}(F) \approx P_{\mathrm{o}}(F) \approx \frac{k_{open}}{\left(k_{open} + k_{close}(F)\right)},$$

where k_{open}/k_{close} are the gate-opening/gate-closing rates, respectively. k_{close} is expected to display a mono-exponential force dependence:

$$k_{close}(F) = k_{close,0} * e^{-\left(F\Delta\mathrm{x}/kT\right)},$$

where $k_{close,0}$ is the closing rate in the absence of tension. Accordingly, at a force of 5 pN, the gate closing rate is:

$$k_{close}(5\ pN) = k_{close,0} * e^{-\left(2.2\ nm*5\ pN/kT\right)} \approx k_{close,0}/15,$$

under the assumption that the transition state length determined for *Ec*TopoI-ssDNA ($\Delta x \approx 2.2$ nm) is the same for TRR-ssDNA. If we also assume that, at 5 pN, at least 90% of the TRR-ssDNA gates are open (as suggested from, e.g., Fig. 2c), it follows that the probability of gate opening at 5 pN is:

$$P_{\mathrm{O}}(5\ pN) = 90\% \approx \frac{k_{open}}{\left(k_{open} + k_{close,0}/15\right)}$$

from which we deduce:

$$k_{close,0} \approx \frac{5}{3} k_{open}$$

Thus, we conclude that the probability of gate opening in the absence of tension is:

$$P_O(0\ pN) = \frac{k_{open}}{(k_{open} + k_{close,0})} \approx \frac{3}{8} \approx 38\%$$

Note that this value is only valid if P_o, i.e., the opening/closing substep, is rate limiting. However, if we instead assume that the opposite is true (i.e., the cleavage/religation substep is rate-limiting), it follows that $P_o \approx 100\%$. In this case, the overall probability of gate opening at 5 pN is: $P_O(5\ pN) \approx P_c(5\ pN)$. Given that 90% of the gates are open at 5 pN (see above) and that we assume no force-dependence of the cleavage/religation substep, 90% of the gates would be open even in the absence of force. Thus, the estimated probability in the case where the opening/closing substep is rate limiting (38%) is a lower bound for the overall fraction of open gates in the absence of tension. Our conclusion that many TRR-ssDNA gates are open at very low forces is further supported by the fact that (i) after stretching TRR-ssDNA in the presence of T-DNA, substantial T-DNA unbinding does not occur as the tension is reduced and (ii) long dsDNA molecules readily interact with TRR-ssDNA even at tensions <2 pN (e.g., Supplementary Fig 4e).

The fact that TRR-ssDNA appears to exhibit substantial gate opening at low tensions is in contrast to both *Ec*TopoI-ssDNA and *Ec*TopoIII-ssDNA. In the latter cases, either the gate closing/religation rate is more than two orders of magnitude larger than the opening/cleavage rates, respectively, in the absence of tension[4]. Thus, either P_o or P_c is <1%, and the total opening probability for both enzymes is <1% at 0 pN. For this reason, substantial gate opening for *Ec*TopoI-ssDNA and *Ec*TopoIII-ssDNA only occurs at forces above ~10 pN (see, for example, Fig. 5b).

Supplementary Note 5: Estimation of the number of T-DNA molecules bound to TRR-ssDNA under maximum coverage conditions

In order to estimate the maximum coverage of circular dsT-DNA on TRR-ssDNA, we first quantified the fluorescence intensity of a single dsT-DNA molecule bound to a TRR-ssDNA substrate (under low dsT-DNA coverage conditions, see Supplementary Fig. 3c). Single dsT-DNA molecules appear elongated along the axis of the tethered ssDNA in the fluorescence image. The average full-width-half-maximum (FWHM) of the fluorescence spot associated with a single dsT-DNA molecule in the direction along the ssDNA substrate was ~530 nm, compared to ~375 nm in the perpendicular direction (Supplementary Fig. 3e). This is because a long dsT-DNA molecule will make simultaneous contact with many TRR-ssDNA gates and thus spread out along the substrate. A deconvolution of the FWHM associated with the parallel and perpendicular Gaussian distributions reveals that the average length that a single dsT-DNA molecule occupies on the ssDNA substrate is ~450 nm. Since the measured end-to-end length (at ~5 pN) of a tethered ssDNA molecule with maximum TRR coverage is typically ~30 µm (Fig. 3b), we estimate that up to ~67 dsT-DNA molecules can interact simultaneously with the TRR-ssDNA substrate (i.e., 30 µm / 0.45 µm). For comparison, we compared the *total* intercalator fluorescence intensity of a TRR-ssDNA substrate containing the maximum dsT-DNA coating achievable under our conditions with that of a TRR-ssDNA substrate containing a single bound dsT-DNA molecule (Supplementary Fig. 3c, d). This indicates a maximum coating of ~100 dsT-DNA molecules. While this is larger than that predicted based on the FWHM measurements (see above), we note that a single dsT-DNA molecule will likely spread out over a broader distance in the absence of other bound dsT-DNA molecules. This will likely cause a slight under-estimate of the maximum number of bound dsT-DNA molecules possible. Nonetheless, our analysis strongly suggests that after 30 seconds incubation in dsT-DNA, the TRR-ssDNA substrate was fully coated with dsT-DNA. We come to similar conclusions when using linear, rather than circular, dsT-DNA (Supplementary Fig. 3f).

Using the procedure described above, we also estimate that ~300 ssT-DNA molecules bind to the TRR-ssDNA substrate under saturation conditions. While it might seem surprising that this is about 3-

times the number obtained for a slightly shorter dsT-DNA substrate, this can be accounted for by the fact that ssDNA has a much lower persistence length compared with dsDNA, and thus it will cover a much shorter distance on the TRR-ssDNA substrate.

Supplementary Note 6: Long T-DNA molecules bind strongly to open gates due to the chelate effect

We observed that long T-DNA molecules exhibit a much higher affinity for the TRR-ssDNA substrate compared to short T-DNA oligos. This is similar to what in coordination chemistry is referred to as the chelate effect, where a complexing agent with multiple binding sites (a so-called multi-dentate ligand) can display binding affinities orders of magnitude higher than those reported for the constituent mono-dentate ligands. The simple explanation for this higher affinity is that the binding of the multi-dentate ligand is entropically favored in that it can only unbind if all constituent binding sites lose contact with the substrate simultaneously. By analogy, in the case of long T-DNA, it is very unlikely that all binding sites (namely the TRR-ssDNA gates) will simultaneously lose contact with the target (i.e., the T-DNA molecule).

Supplementary Note 7: dsT-DNA can compete with ssT-DNA for binding to TRR-ssDNA

As shown in Fig. 3a, dsT-DNA and ssT-DNA (each bound to TRR-ssDNA) can be efficiently stained by intercalator dyes when using the standard buffer (Methods). The ability of these dyes to stain ssT-DNA is most likely due to segments of ssT-DNA (that are not interacting directly with the TRR-ssDNA) forming hairpin structures. However, this makes it difficult to differentiate between dsT-DNA and ssT-DNA when both are bound to the TRR-ssDNA substrate simultaneously. Since hairpin formation is less favoured under low salt conditions, we tested whether intercalator dye can be prevented from binding to ssT-DNA in melting buffer (Methods).

To this end, we incubated a tethered ssDNA substrate in TRR-mCherry, followed by ssT-DNA (both in standard buffer) before moving the substrate to a channel containing melting buffer and intercalator dye. In this latter channel, we observed TRR-mCherry fluorescence as well as substantial intercalator fluorescence, indicating that low salt alone was not sufficient to disrupt hairpin formation on bound ssT-DNA (Supplementary Fig. 4d). We next re-incubated the same ssT-TRR-ssDNA substrate in the channel containing TRR-mCherry and observed a greatly increased TRR-mCherry fluorescence signal, which we attribute to the binding of free TRR to segments of ssT-DNA that were not directly interacting with the TRR-ssDNA substrate. Finally, we re-incubated this substrate in the channel containing melting buffer and intercalator dye and observed much less intercalator fluorescence than we did on the first incubation in the same channel. Based on these findings, we propose that the *combined* effect of low salt and TRR binding to ssT-TRR-ssDNA inhibits the ability of the ssT-DNA to form hairpin structures. As a result, there is negligible binding of intercalator dye to ssT-DNA under these conditions.

The above approach was then used to differentiate between dsT-DNA and ssT-DNA when both are bound to TRR-ssDNA simultaneously. To demonstrate this, we repeated the above procedure, with the only difference that we used an equimolar mixture of long dsT-DNA and ssT-DNA rather than ssT-DNA alone. In this case, substantial intercalator fluorescence was still detected in the last incubation step, much higher than that observed when using only ssT-DNA. We therefore conclude that a substantial fraction of dsT-DNA was bound to the TRR-ssDNA substrate, alongside ssT-DNA.

Supplementary Note 8: dsT-DNA also binds to TRR-ssDNA at very low tensions

In our standard dsT-binding experiments, we transiently applied tension to the tethered ssDNA substrate when incubating in TRR (~15 pN) and T-DNA (~5 pN) (Methods). In order to demonstrate that this tension did not influence T-DNA binding, we performed the following control experiment. First, we incubated a bare ssDNA molecule in TRR at less than 5 pN. TRR binding induced a decrease of the force to less than 2 pN (resulting in negligible force on the tethered substrate). Under these conditions, we observed considerable binding of TRR to the tethered ssDNA (Supplementary Fig. 4e), although the coverage was somewhat reduced compared to standard experiments (Fig. 3a). The reduced TRR coverage is attributed to the fact that at low (<5 pN) tensions, the formation of dsDNA hairpins in the tethered ssDNA may suppress TRR binding. The TRR-ssDNA was then incubated in dsT-DNA and subsequently imaged in a buffer containing intercalator dye, all the while maintaining the tension at less than 2 pN. The corresponding intercalator fluorescence image (Supplementary Fig. 4e) revealed efficient dsT-DNA binding, indicating that the latter does not require the application of substantial tension.

**Figure**	**Ch III**	**Ch IV**	**Ch V**	**Ch VI**
2a,b,d,f / 5a / S2a,b,d,e / S3b / S6b	*S*	*M*	TRR or *Ec*TopoI + *S*	*E*
2e, S6a	*S*	*M*	TRR or *Ec*TopoI + *E*	*S*
3a-d / 5c,e,f / S3b-f / S4a(*right*),b,c,e	*M*	TRR + *S*	dsT or ssT + *S*	I + *S*
4a-e/ 5d,g / S5a-d	*M*	dsT or ssT + *S*	TRR or *Ec*TopoI + *S*	I + *H*
6a,b / S7a-d	*S*	*M*	TRR + *S*	BLM + *S*
6d,e / S7e,f	*M*	dsT + *S*	TRR + BLM + *S*	I + *S*
S2c	*S*	*M*	TRR-Y337F + *S*	*S*
S3a	*S*	*M*	TRR + *S*	ss/ds-oligo + *S*
S4a (*left*)	*M*	*S*	TRR + *S*	dsT + I + *S*
S4d	*M*	TRR + *S*	dsT (or ssT + dsT) + *S*	I + *M*

Supplementary Table 1. Overview of buffer conditions in different channels of the flow cell used to obtain the data presented in Figs. 2-6 and Supplementary Figs. 2-7. The flow cell contained 6 channels, numbered I-VI (see Supplementary Fig. 1). Here, *S*, *M* and *H* indicate the standard buffer, melting buffer and high salt buffer, respectively, as defined in Methods. *E* denotes the standard buffer without magnesium, supplemented with 1 mM EDTA. I represents intercalator dye. The concentration of TRR, BLM, T-DNA and intercalator dye was as described in Methods. Channel I contained streptavidin-coated polystyrene beads in standard buffer: the bead diameter was 4.65 μm, with the exception of Fig. 2d, where it was 1.75 μm. Channel II contained biotin-labeled λ-DNA in standard buffer, with the exception of Fig. 2d, where biotin-labeled pKYB1-DNA was used. Force-induced melting of duplex λ-DNA / pKYB1-DNA was performed in melting buffer (in either channel III or IV).

Supplementary Figures

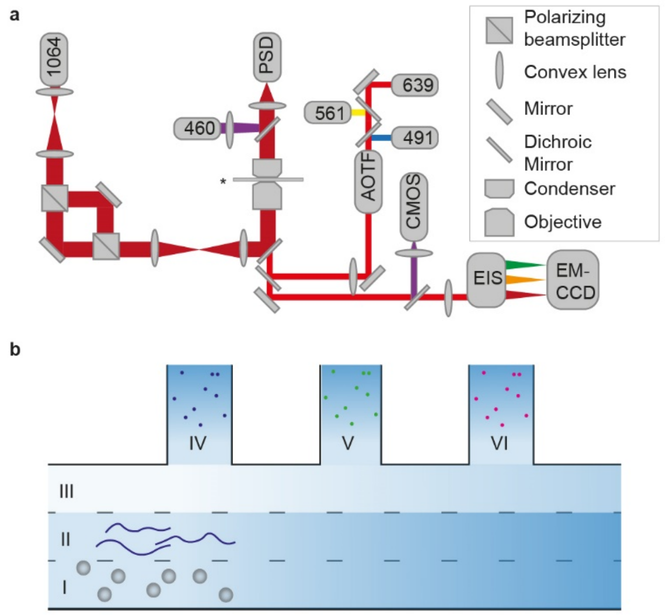

Supplementary Fig. 1: Experimental setup. a Beam paths and relevant optical components[3]. The dark, thick red lines indicate the infrared laser beam path used to create double optical tweezers and measure applied forces. The lighter, thin red lines depict the beam paths for bright-field illumination as well as fluorescence excitation and detection. The asterisk indicates the location of the flow cell. A detailed description of the setup is provided in Methods. Abbreviations: 1064 = 1064 nm laser for optical trapping; PSD = position-sensitive diode; 460 = 460 nm LED for bright-field illumination; AOTF = acousto-optical tunable filter; 491/561/639 = 491/561/639 nm lasers for fluorescence excitation; CMOS = Complementary Metal-Oxide Semiconductor camera for bright-field detection; EIS = emission-image splitter; EM-CCD = Electron-Multiplying Charge-Coupled Device camera for fluorescence detection. **b** Schematic representation of the multichannel flow-cell containing 6 channels. Two of the channels (I and II) are used to catch beads and DNA, respectively, while the other four channels (III–VI) can be used to expose the tethered DNA molecule to different buffer and/or protein conditions, as outlined in Methods and Supplementary Table 1.

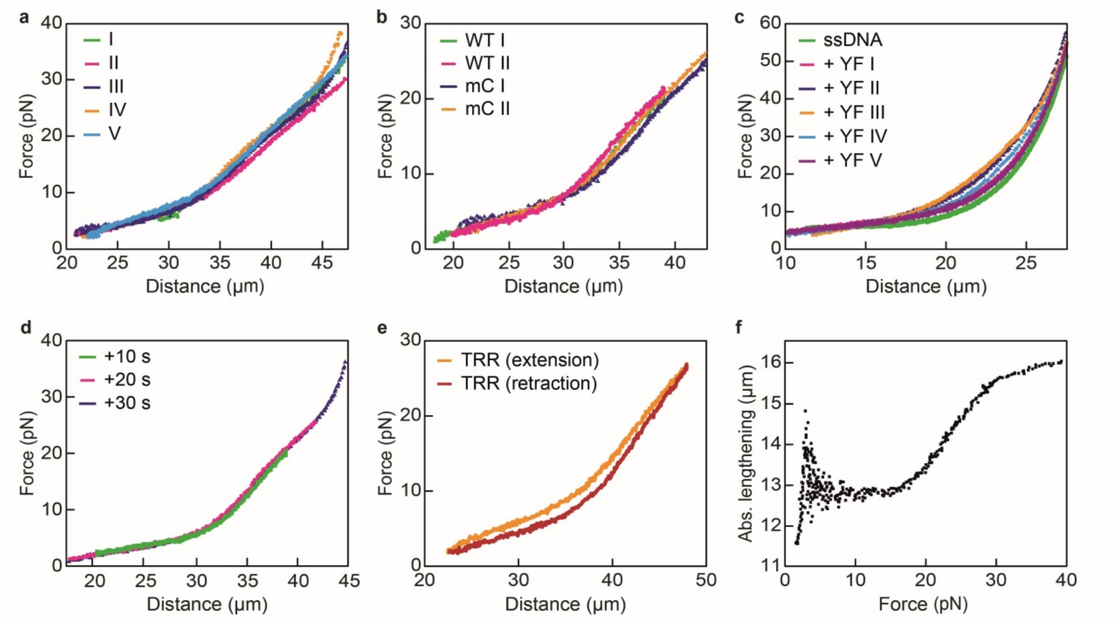

Supplementary Fig. 2: Overview of TRR-ssDNA gate opening mechanics. a Comparison of FD-curves from five different TRR-ssDNA substrates (I-V) shows that the force-extension characteristics are highly reproducible. **b** Representative TRR-ssDNA FD-curves associated with unlabeled TRR (WT I, WT II) and mCherry-labeled TRR (mC I, mC II), from at least 10 independent measurements. These curves show very similar features, demonstrating that mCherry labeling does not significantly affect the biophysical properties of TRR binding to ssDNA. **c** Five different FD-curves of ssDNA coated with a catalytically-dead TRR mutant (YF I-V). For reference, the FD-curve of bare ssDNA is shown in green. These curves show that the catalytically-dead TRR mutant does not result in significant lengthening of bare ssDNA. **d** Three consecutive FD-curves recorded for the same TRR-ssDNA substrate exhibit similar behaviours. Given that the movement of the tethered ssDNA substrate into the TRR-containing channel took less than 10 seconds, we conclude that a) TRR binding, b) backbone cleavage and c) gate opening must be completed on timescales much faster than that, most likely on the order of 1 second or less. In support of this, we note that backbone cleavage and gate opening have been reported to occur on similar timescales for *Ec*TopoI[4]. **e** Representative extension (orange) and retraction (red) FD-curve of TRR-ssDNA (from $N \geq 20$) showing minimal hysteresis. **f** Subtraction plot showing the absolute (abs.) lengthening of ssDNA due to TRR (calculated using the FD-curves shown in Fig. 2b, as described in Methods). This plot is identical to that in Fig. 2c with the exception that, here, data below 5 pN are also shown and the relative lengthening is not normalized. Source data are provided as a Source Data file.

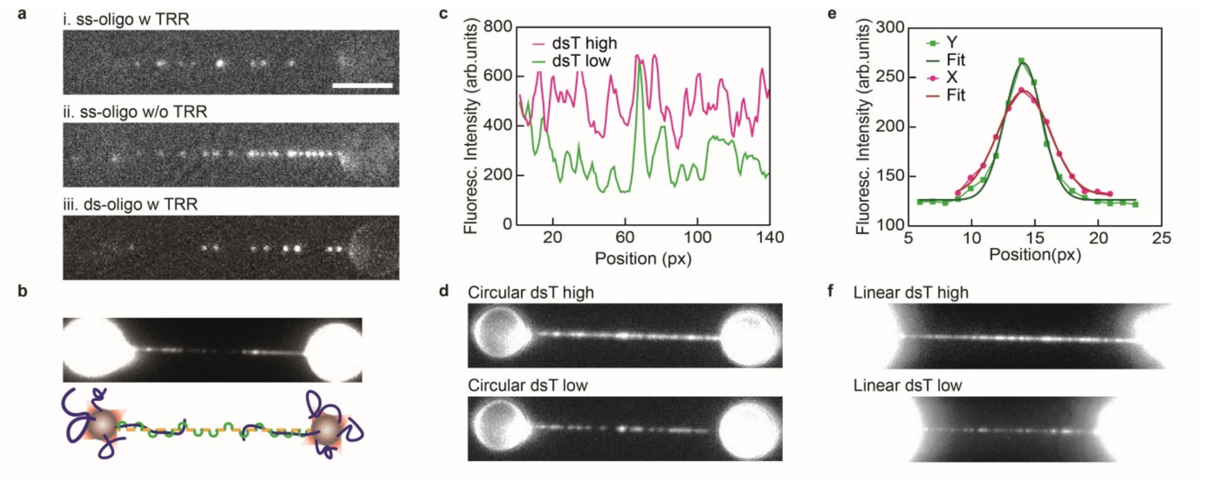

Supplementary Fig. 3: Interaction of TRR-ssDNA with T-DNA. a Fluorescence images of short (30 nt/bp) oligonucleotides of Atto647N-labeled ssDNA (***i***,***ii***) and Atto647N-labeled dsDNA (***iii***) bound, respectively, to tethered ssDNA with (w, ***i***,***iii***) or without (w/o, ***ii***) TRR coating, from at least 5 independent measurements. The number of bound ssDNA and dsDNA oligonucleotides (<50 molecules) observed in (***i***) and (***iii***) is negligible relative to the TRR coating (>>1000 proteins, see Supplementary Note 2). Furthermore, the observed oligonucleotide binding events can most likely be accounted for by non-specific binding of the oligomers to regions of bare ssDNA. Scale bar represents 5 μm and applies to all fluorescence images in the figure. **b** Intercalator fluorescence image of TRR-ssDNA bound by surplus, bead-tethered λ-dsDNA molecules, from at least 10 independent measurements. In the case shown here, intercalator fluorescence is observed at both ends of the TRR-ssDNA substrate, indicating that dsDNA molecules from both beads are interacting with the TRR-ssDNA substrate, as illustrated schematically below the image. **c** Intercalator fluorescence (fluoresc.) intensity associated with the images in panel (**d**), corresponding to low and high coverage of long dsDNA (4,361 bp) on the TRR-ssDNA substrate. The position along the tethered TRR-ssDNA is indicated in pixels (px, ~130 nm). **d** (Top) Intercalator fluorescence image after 30 seconds incubation of TRR-ssDNA with long circular dsDNA, showing a strong and homogenous coating of dsT-DNA, from at least 10 independent measurements. (Bottom) Intercalator fluorescence image after a brief (<5 seconds) incubation of TRR-ssDNA with long circular dsDNA, from 2 independent measurements. Only a limited number of bound dsDNA molecules was observed. **e** Intercalator fluorescence intensity of a single long dsDNA molecule bound to the TRR-ssDNA substrate, in both the parallel (x) and perpendicular (y) directions with respect to the TRR-ssDNA molecular axis, obtained under low dsDNA coverage conditions. Note that the spot size in the x-direction was significantly larger than the corresponding distribution in the y-direction, indicating that the dsDNA spreads out along the TRR-ssDNA substrate (Supplementary Note 5). **f** Intercalator fluorescence images of TRR-ssDNA bound by long linearized

dsT-DNA molecules, from at least 5 independent measurements. Similar to the case of long circular dsT-DNA (panel (**d**)), images were obtained at high (top) and low (bottom) dsT-DNA coating. Source data are provided as a Source Data file.

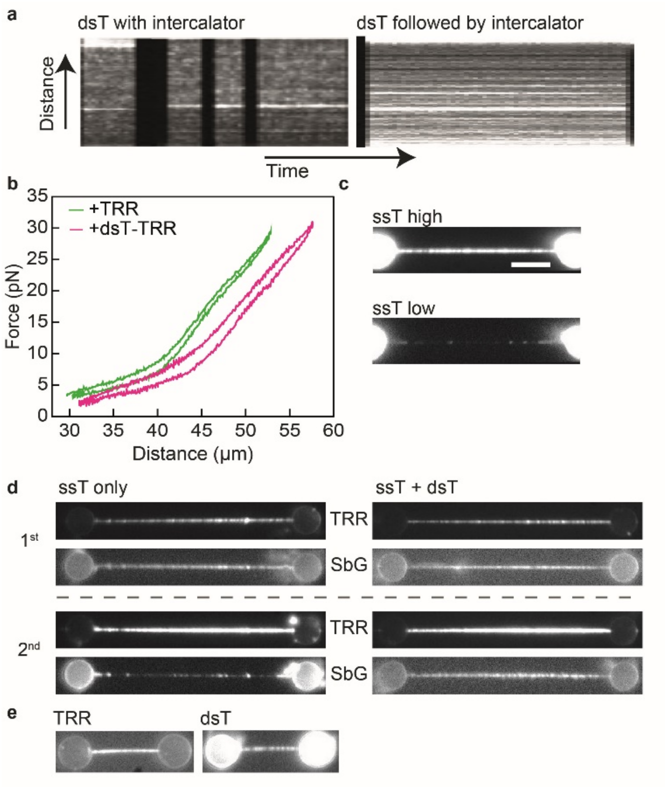

Supplementary Fig. 4: Binding of dsT- and ssT-DNA to TRR-ssDNA. a (Left) Representative kymograph (from $N = 3$) of intercalator fluorescence for a TRR-ssDNA substrate incubated in a solution containing both long dsT-DNA and intercalator dye (kymograph obtained from Supplementary Movie 1). The majority of the dsT-DNA molecules do not bind stably to the TRR-ssDNA substrate (thus the kymograph displays a blurred pattern, corresponding to transient dsT-DNA binding events). (Right) Representative kymograph (from $N \geq 10$) of intercalator fluorescence for a TRR-ssDNA substrate that was incubated first in a solution of long dsT-DNA and *then* in a solution of intercalator dye. Note that, in this case, dsT-DNA binding events (horizontal lines) of much longer durations were observed than in the case of the left panel (where the dT-DNA and intercalator were in the same channel). **b** Comparison of FD-curves (extension and retraction, from $N \geq 10$) for TRR-ssDNA in the absence (green) and presence (pink) of long dsT-DNA, indicating no significant change in the hysteresis between the two cases. **c** Representative intercalator fluorescence images for a TRR-ssDNA molecule with a high (top, from $N \geq 10$) and low (bottom, from $N = 4$) coating of long ssT-DNA, respectively. Scale bar is 5 μm and applies to all snapshots. **d** dsT-DNA can compete with ssT-DNA for binding to TRR-ssDNA. (Top left) mCherry and intercalator fluorescence images for a TRR-ssDNA substrate (after the 1st incubation of ssDNA in TRR) bound by long ssT-DNA, imaged in melting buffer containing intercalator dye. The substantial intercalator fluorescence observed indicates hairpin formation in regions of the ssT-DNA. (Bottom left) mCherry and intercalator fluorescence images obtained for the same tether as shown above, but after a 2nd

incubation in TRR-mCherry (in standard buffer), and followed by re-incubation of the substrate in melting buffer containing intercalator dye. The increased mCherry fluorescence reflects additional TRR binding to free sections of the ssT-DNA, while the decreased intercalator fluorescence indicates that hairpin formation in the bound ssT-DNA is much lower than after the 1st TRR incubation (see above). (Top right) mCherry and intercalator fluorescence images for a TRR-ssDNA substrate (after the 1st incubation of ssDNA in TRR) bound by an equimolar mixture of long dsT-DNA and ssT-DNA, imaged in melting buffer containing intercalator dye. (Bottom right) mCherry and intercalator fluorescence images obtained for the same tether as shown above but after the 2nd incubation in TRR-mCherry (in standard buffer) followed by re-incubation of the substrate in melting buffer containing intercalator dye. In contrast to the case where the T-DNA was only single-stranded (bottom left), substantial intercalator fluorescence signal was observed, indicating that dsT-DNA was also bound abundantly to the TRR-ssDNA substrate. Representative data are shown from at least 4 independent measurements. **e** dsT-DNA can bind to TRR-ssDNA even at very low tensions (<2 pN). (Left) mCherry fluorescence image of a tethered ssDNA molecule incubated in TRR-mCherry at <5pN. (Right) Intercalator fluorescence image of the same TRR-ssDNA tether after incubation in long dsT-DNA at <2 pN (revealing substantial dsT-DNA binding). Representative data are shown from 3 independent measurements. Source data are provided as a Source Data file.

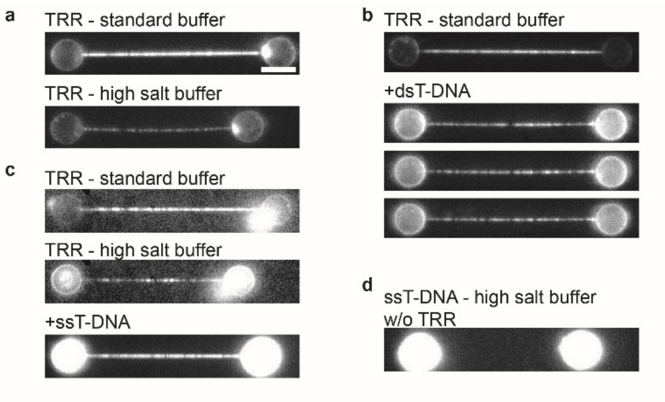

Supplementary Fig. 5: Catenation of T-DNA to ssDNA. a mCherry fluorescence images (from $N \geq 20$) from the same TRR-ssDNA substrate as shown in Fig. 4c before (top) and after (bottom) incubation in high-salt buffer. The scale bar represents 5 µm, and applies to all snapshots in this figure. **b** mCherry fluorescence (top) and intercalator fluorescence (bottom) image (from $N \geq 10$) for a tethered TRR-ssDNA molecule that was incubated in a solution of circular dsT-DNA molecules. All incubation and imaging was performed in standard buffer. Note that the TRR coating on the tethered ssDNA was lower here than that in panel (**a**). The images in the bottom three panels correspond to intercalator fluorescence recorded at different time intervals: the changing fluorescence pattern over time (corresponding to frames from Supplementary Movie 3) indicates that the circular dsT-DNA is catenated to the tethered ssDNA substrate. **c** (Top) mCherry fluorescence from a TRR-ssDNA substrate coated with circular ssT-DNA in standard buffer. (Middle) mCherry fluorescence from the same substrate as in the upper image but after moving to a high salt buffer (TRR unbinds). (Bottom) Corresponding intercalator fluorescence in the high-salt buffer. Representative data are shown from at least 5 independent measurements. **d** Intercalator fluorescence image of a tethered ssDNA molecule (not bound by TRR) in a high-salt buffer containing intercalator dye following incubation in a solution of circular ssT-DNA. Representative data are shown from 2 independent measurements. Source data are provided as a Source Data file.

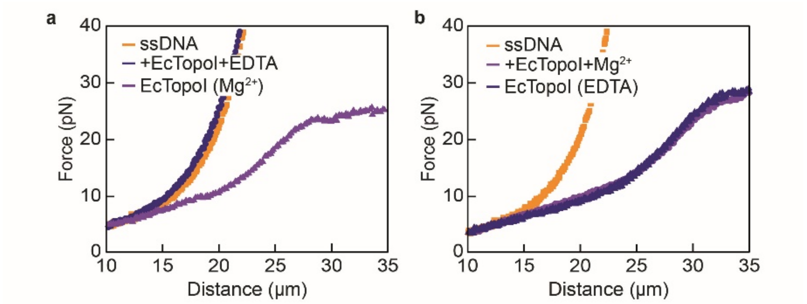

Supplementary Fig. 6: Effect of magnesium on *Ec*TopoI-ssDNA. a Representative FD-curves (from $N \geq 10$) of bare ssDNA (orange); ssDNA after incubation in a Mg^{2+}-deficient buffer containing *Ec*Topo1 (dark blue); and the same tethered ssDNA substrate (bound by *Ec*Topo1) after moving to the standard buffer, containing Mg^{2+} (purple). **b** Representative FD-curves (from $N \geq 5$) of bare ssDNA (orange); ssDNA after incubation in standard buffer containing *Ec*Topo1 (purple); and the same tethered ssDNA substrate (bound by *Ec*Topo1) after moving to a Mg^{2+}-deficient buffer (dark blue). Note that when moving the *Ec*TopoI-ssDNA substrate from the channel containing standard buffer to the Mg^{2+}-deficient buffer, we applied a force of >10 pN in order to keep most of the *Ec*Topo1-ssDNA gates in the open (and thus also cleaved) state[4]. For TRR-ssDNA, this is not required, as most of the bound TRR are already in the open state at low tensions (Supplementary Note 4). Source data are provided as a Source Data file.

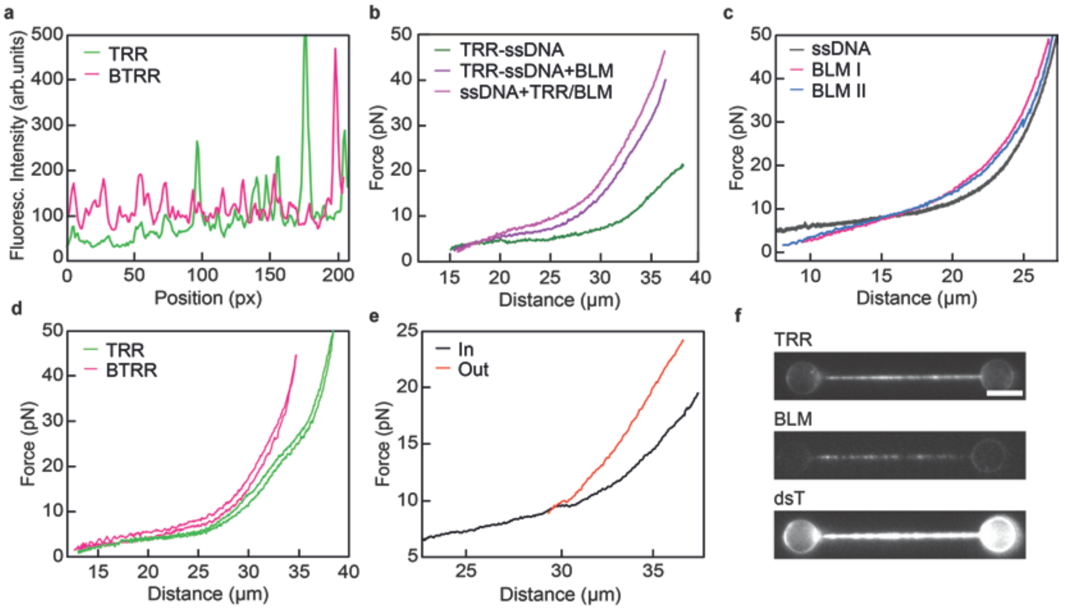

Supplementary Fig. 7: Interaction of BTRR with tethered ssDNA. a Comparison of the mCherry fluorescence (fluoresce.) intensity from, respectively, TRR-ssDNA (green) and BTRR-ssDNA (pink), indicating that a similar or slightly higher TRR coating was obtained in the presence of BLM. The position along the tethered substrate is indicated in pixels (px, ~130 nm). **b** Representative FD-curves (from $N = 2$) of TRR-ssDNA (green) and BTRR-ssDNA (pink and purple). The FD-curve of TRR-ssDNA shows the expected features, including substantial lengthening (relative to bare ssDNA) and a 'shoulder' at ~15pN. The FD-curves of BTRR-ssDNA were generated via two different procedures. In one procedure, the FD-curve was obtained by incubating TRR-ssDNA in a solution of BLM (purple), while in the other, the FD-curve was derived by incubating ssDNA in an equimolar mixture of BLM and TRR (pink). In both procedures, the FD-curves of BTRR-ssDNA displays greatly reduced lengthening compared with that of TRR-ssDNA and do not exhibit a 'shoulder' at 15 pN. Note that the green and purple FD-curves were both obtained using the same molecule. **c** Two FD-curves of BLM-ssDNA (BLM I and II), demonstrating reproducible stretching characteristics (for reference, the bare ssDNA curve is shown in grey). **d** Comparison of FD-curves (extension and retraction) of TRR-ssDNA (green) and BTRR-ssDNA (pink, from $N \geq 5$), indicating no significant change in the hysteresis (associated with the extension and retraction curves) between the two cases. **e** Comparison of FD-curves (from $N = 4$) of dsT-BTRR-ssDNA substrates recorded outside (red) and inside (black) the channel containing free dsT-DNA. This reveals a slight length reduction when the substrate is stretched outside, rather than inside, the dsT-DNA channel. We interpret this as follows: a fraction of the dsT-DNA molecules on the BTRR-ssDNA substrate are bound transiently and therefore readily detach when moved to a buffer-only channel. Since we did not observe this for dsT-TRR-ssDNA, we propose that BLM partially destabilizes the interaction of dsT-DNA with TRR-ssDNA.

Note that it was generally not possible to visualize dsT-DNA inside the dsT-DNA channel because, as shown in Supplementary Fig. 4a, the presence of intercalator dye interferes with the interaction of dsT-DNA with TRR-ssDNA. **f** Fluorescence images (from $N \geq 5$) of a tethered ssDNA molecule following incubation in a channel containing TRR, BLM and long dsT-DNA, imaged using mCherry, Snap649 and intercalator dye fluorescence, respectively. Note that in this case, the dsT-DNA concentration was significantly higher compared to the example in Fig. 6d, and resulted in a greatly reduced binding of BLM. Scale bar is 5 µm. Source data are provided as a Source Data file.

References

1. Ke, C., Humeniuk, M., S-Gracz, H. & Marszalek, P. E. Direct measurements of base stacking interactions in DNA by single-molecule atomic-force spectroscopy. *Phys. Rev. Lett.* **99**, 018302 (2007).
2. Saenger, W. *Principles of Nucleic Acid Structure*. Springer, New York (1984).
3. Sarlós, K. et al. Reconstitution of anaphase DNA bridge recognition and disjunction. *Nat. Struct. Mol. Biol.* **25**, 868–876 (2018).
4. Mills, M., Tse-Dinh, Y. C. & Neuman, K. C. Direct observation of topoisomerase IA gate dynamics. *Nat. Struct. Mol. Biol.* **25**, 1111–1118 (2018).
5. Champoux, J. J. DNA Topoisomerases: structure, function, and mechanism. *Annu. Rev. Biochem.* **70**, 369–413 (2001).
6. Bocquet, N. et al. Structural and mechanistic insight into Holliday-junction dissolution by Topoisomerase IIIα and RMI1. *Nat. Struct. Mol. Biol.* **21**, 261–268 (2014).
