# Supplementary material for: Duplex DNA and BLM regulate gate opening by the human TopoIIIα-RMI1-RMI2 complex
Source: Nat Commun. 2022 Jan 31;13:584. doi: 10.1038/s41467-022-28082-5 (PMC8803869; doi:10.1038/s41467-022-28082-5)
Supplement: Supplementary file 3 — Description of Additional Supplementary Files [file 41467_2022_28082_MOESM3_ESM.pdf]

Legends to supplementary datasets, software and movies

Supplementary Data 1: Three additional examples for the data shown in Fig. 2a,e,f.

Supplementary Data 2: Three additional examples for the data shown in Fig. 3a,b.

Supplementary Data 3: Three additional examples for the data shown in Fig. 4a,b,c.

Supplementary Data 4: Three additional examples for the data shown in Fig. 5a,c,d,f,g.

Supplementary Data 5: Three additional examples for the data shown in Fig. 6a,d,e.

Supplementary Software 1: ZIP-folder containing the code written in MATLAB R2015b for the stepfitting algorithm used to fit the data in Fig. 2c, and a brief description.

Supplementary Movie 1: The interaction of dsT-DNA with TRR-ssDNA is destabilized in the presence of intercalator dye. Representative movie showing the intercalator fluorescence over time for a TRR-ssDNA substrate in a solution containing both long dsDNA and intercalator dye. Note that this is the same substrate as that shown in Supplementary Fig. 4a, left. The vast majority of interacting dsDNA molecules do not bind stably and dissociate after a short (second-scale) period of time. Nevertheless, due to the presence of free dsDNA in solution, dissociation of bound dsDNA is quickly followed by transient binding of further dsDNA molecules. To highlight the transient nature of dsDNA binding, the movie is sped up 4-fold. The pixel size is 135 nm.

Supplementary Movie 2: Demonstration of dsT-DNA catenation using high salt buffer to induce TRR unbinding. Representative movie showing intercalator fluorescence after moving a TRR-ssDNA substrate coated with dsT-DNA to a high salt buffer. During the movie, buffer flow is applied in alternating directions along the molecular axis of the tethered ssDNA substrate. Frames from this movie were used to compile Fig. 4c, left. The application of buffer flow demonstrates the high mobility of the dsT-DNA molecules. Together with the fact that most TRR has unbound from the ssDNA in high salt buffer (Supplementary Fig. 5a. data from the same molecule), these findings demonstrate that the dsT-DNA molecules are catenated around the tethered ssDNA. The movie as displayed here is sped up 4-fold, and the pixel size 135 nm.

Supplementary Movie 3: Demonstration of dsT-DNA catenation under standard buffer conditions. Representative movie of intercalator fluorescence for a TRR-ssDNA substrate coated with dsT-DNA in standard buffer (see Supplementary Table 1). Note that a lower TRR coating than that in Supplementary Movie 2 was used. Frames from this movie were used to compile Supplementary Fig. 5b. The majority of bound dsT-DNA molecules visualized here show diffusion on the TRR-ssDNA substrate, consistent with catenation. The movie as displayed here is sped up 4-fold, and the pixel size 135 nm.
